# Simulated full weight bearing following posterior column acetabular fracture fixation: a biomechanical comparability study

**DOI:** 10.1186/s13018-023-03879-2

**Published:** 2023-06-02

**Authors:** Till Berk, Ivan Zderic, Peter Schwarzenberg, Torsten Pastor, Roman Pfeifer, Sascha Halvachizadeh, Geoff Richards, Boyko Gueorguiev, Hans-Christoph Pape

**Affiliations:** 1 AO Research Institute Davos, Clavadelerstrasse 8, 7270 Davos, Switzerland; 2 Department of Trauma, University Hospital Zurich, Raemistrasse 100, 8091 Zurich, Switzerland; 3 Harald-Tscherne Laboratory for Orthopedic and Trauma Research, University of Zurich, Sternwartstrasse 14, 8091 Zurich, Switzerland; 4 Department of Orthopaedic and Trauma Surgery, Cantonal Hospital Lucerne, Lucerne, Switzerland

**Keywords:** Posterior column acetabulum fracture, Revision cup, Plating, Screw fixation, Artificial bone model, Biomechanics, Motion tracking

## Abstract

**Purpose:**

The incidence of acetabular fractures (AFs) is increasing in all industrial nations, with posterior column fractures (PCFs) accounting for 18.5–22% of these cases. Treating displaced AFs in elderly patients is a known challenge. The optimal surgical strategy implementing open reduction and internal fixation (ORIF), total hip arthroplasty (THA), or percutaneous screw fixation (SF), remains debated. Additionally, with either of these treatment methods, the post-surgical weight bearing protocols are also ambiguous. The aim of this biomechanical study was to evaluate construct stiffness and failure load following a PCF fixation with either standard plate osteosynthesis, SF, or using a screwable cup for THA under full weight bearing conditions.

**Methods:**

Twelve composite osteoporotic pelvises were used. A PCF according to the Letournel Classification was created in 24 hemi-pelvis constructs stratified into three groups (n = 8) as follows: (i) posterior column fracture with plate fixation (PCPF); (ii) posterior column fracture with SF (PCSF); (iii) posterior column fracture with screwable cup fixation (PCSC). All specimens were biomechanically tested under progressively increasing cyclic loading until failure, with monitoring of the interfragmentary movements via motion tracking.

**Results:**

Initial construct stiffness (N/mm) was 154.8 ± 68.3 for PCPF, 107.3 ± 41.0 for PCSF, and 133.3 ± 27.5 for PCSC, with no significant differences among the groups, *p* = 0.173. Cycles to failure and failure load were 7822 ± 2281 and 982.2 ± 428.1 N for PCPF, 3662 ± 1664 and 566.2 ± 366.4 N for PCSF, and 5989 ± 3440 and 798.9 ± 544.0 N for PCSC, being significantly higher for PCPF versus PCSF, *p* = 0.012.

**Conclusion:**

Standard ORIF of PCF with either plate osteosynthesis or using a screwable cup for THA demonstrated encouraging results for application of a post-surgical treatment concept with a full weight bearing approach. Further biomechanical cadaveric studies with larger sample size should be initiated for a better understanding of AF treatment with full weight bearing and its potential as a concept for PCF fixation.

## Introduction

Acetabular fractures (AFs) in the older population are with increasing incidence in all industrial nations and represent the most rapidly growing section of acetabular trauma [1–3]. Posterior column fractures (PCFs) account for up to 18.5–22% of all AFs [4, 5]. Treating displaced AFs in elderly patients is knowingly challenging [6] since achieving anatomic reduction can be tremendously difficult in presence of articular surface impaction, bone loss, and fracture comminution. The surgical treatment via total hip arthroplasty (THA) makes the anatomical reconstruction secondary, while potentially creating other surgical challenges such as prosthetic component (cup) loosening or dislocation of the hip [7]. The optimal strategy of implementing either open reduction and internal fixation (ORIF) or THA remains controversial [8, 9]. The standard procedure for ORIF is plate osteosynthesis. A minimally invasive approach could be percutaneous screw osteosynthesis. Following ORIF of AFs, partial weight bearing for several weeks post operation is a standard procedure. In fact, full weight bearing restriction for 10–12 weeks, followed by an additional period of progressively increasing weight bearing, is thought to be the gold standard postoperative procedure for the majority of orthopaedic trauma surgeons [10]. It is also believed that full weight bearing in the early postoperative phase can endanger the reconstruction stability and therefore the outcome [11]. However, early postoperative movement and patient mobilization lead to higher functional scores and less impaired muscle torque [12, 13]. Furthermore, early recovery of mobilization results in lower complication rates, shorter length of hospital stay, higher autonomy, and reduced mortality [14, 15]. To our knowledge, studies on postoperative full weight bearing following AFs are very scarce and have poor comparability due to heterogeneous fracture personalities and patient morphologies as well as difficult-to-compare implants and surgical techniques.

Purpose: The aim of this biomechanical study was to evaluate construct stiffness and failure load as measures of axial force resistance and primary stability of PCF fixation with either standard plate osteosynthesis, screw osteosynthesis, or using a screwable cup for THA under full weight bearing conditions.

## Materials and methods

### Specimens and preparation

**Fig. 1:**
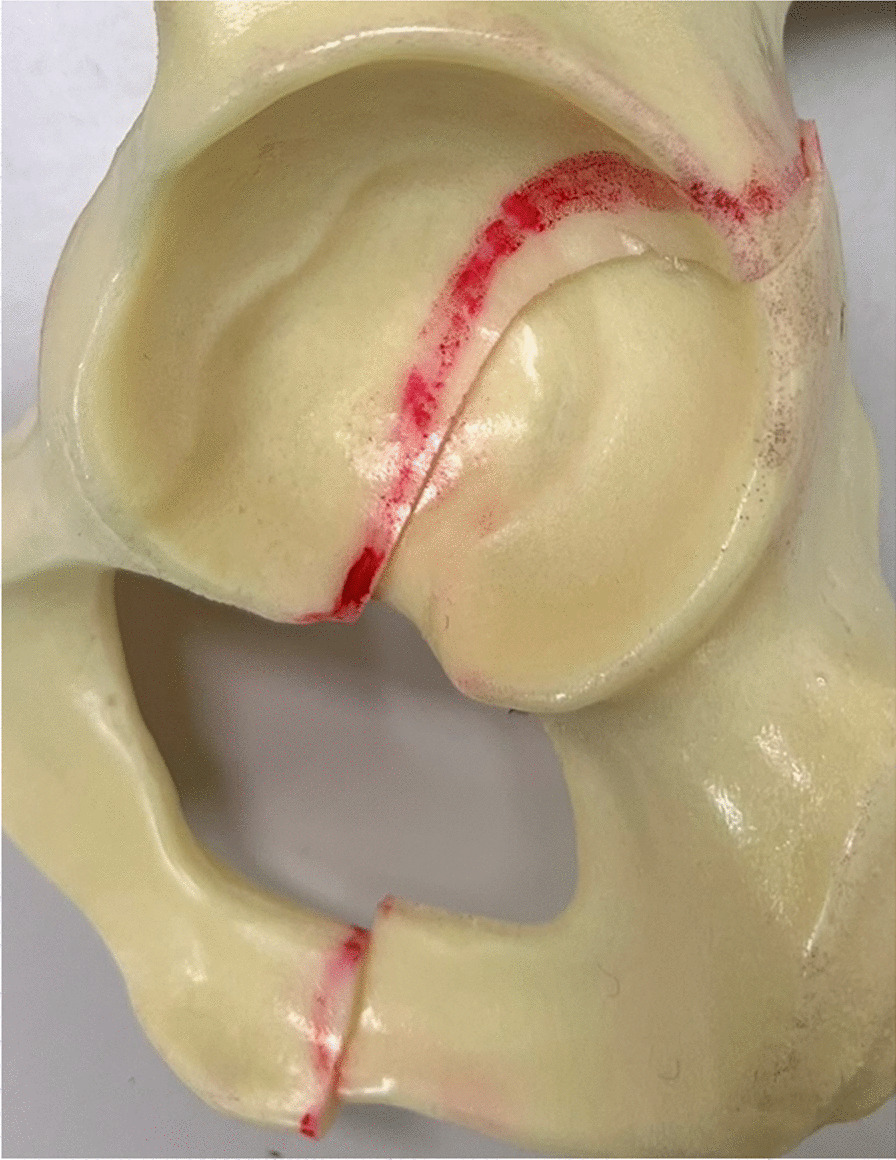
Posterior column acetabular fracture

Twelve composite osteoporotic pelvises were used in this study (Model LSS4055®, Synbone, Zizers, Switzerland). A PCF according to the Letournel Classification [16] was created by means of an osteotomy using a 1 mm bone sawblade and a custom cutting guide made of polymethylmethacrylate (PMMA, SCS-Beracryl D-28, Suter Kunststoffe AG/Swiss-Composite, Fraubrunnen, Switzerland) to ensure that all osteotomies were identical (Fig. 1). Each pelvis was considered for preparation and biomechanical testing of its both left and right sides, resulting in 24 hemi-pelvis constructs, which were stratified into three groups of eight specimens each (n = 8) for instrumentation as follows. In group "posterior column plate fixation" (PCPF), the fracture was treated using a standard 3.5 mm 8-hole stainless steel (316LVM) reconstruction plate (DePuy Synthes, Zuchwil, Switzerland). In group, "posterior column screw fixation" (PCSF), the fracture was treated with a single partially threaded 7.3 mm stainless steel (316LVM) cannulated screw, 90 mm in length (DePuy Synthes, Zuchwil, Switzerland). In group "posterior column screwable cup" (PCSC), the fracture was treated using a cementless, press fit, screw augmented, 58 mm Mpact Two-hole acetabular shell with two cancellous bone screws, 6 mm and 5 mm in diameter, 90 mm and 50 mm in length (Medacta International, Strada Regina, Switzerland).

Anatomical reduction in all PCFs was achieved with the aid of two supraacetabular Weber reduction clamps and two supraacetabular Kirschner (K-) wires placed anteroposteriorly and posteroanteriorly. All procedures were performed by an experienced surgeon following the surgical guidelines of the implant manufacturers, and the AO surgery references and recommendations in order to minimize the interobserver variability [17].

Plate fixation began with contouring each plate according to the anatomical geometry of the specimens, followed by attachment to the bone using reduction clamps. The contouring ensured that the plate did not protrude from the bone at any location. Next, three bicortical screws of appropriate length were placed in both the most proximal and distal holes under direct visualization to achieve reliable fixation. Care was taken that the corresponding screws were oriented in the same direction within all specimens of this group. For increased plate stability, the most cranial screw of the distal screw cluster was placed interfragmentary through the dorsal part of the acetabular corridor.

For the screw fixation, following anatomical PCF reduction, a 2.8 mm guide wire was inserted across the fracture and cephalad through the proximal region of the inferior pubic ramus in an antegrade fashion. The entry point for K-wire placement was radiologically determined at the posterior portion of the ilium crest. During guide wire placement, continuous monitoring under fluoroscopy was performed to avoid any perforations, via falsa, or cortical disruptions. Following pilot drilling, each cannulated screw was placed over the K-wire and tightened according to the operator's best practice.

For the cup implantation, the acetabulum was reamed in stages, starting with a 46 mm reamer and ending with  a 58 mm one. All cups were implanted with gentle hammer blows in an ideal position regarding inclination and anteversion. The two holes in the cranial aspect of the cup allowed its fixation with screws in the superior dome. For the PCF treatment, the cup was rotated clockwise by 45° with respect to its standard position, such that one screw could be positioned superiorly and one screw—inferiorly to the fracture site. With this orientation, the two screws stabilized the cup, bridging itself the fracture. The cranial screw could be inserted monocortically in the direction of the acetabular dome with a length of 90 mm. The caudal screw was placed bicortically in the posteroinferior quadrant of the acetabulum with a length of 50 mm.

**Fig. 2:**
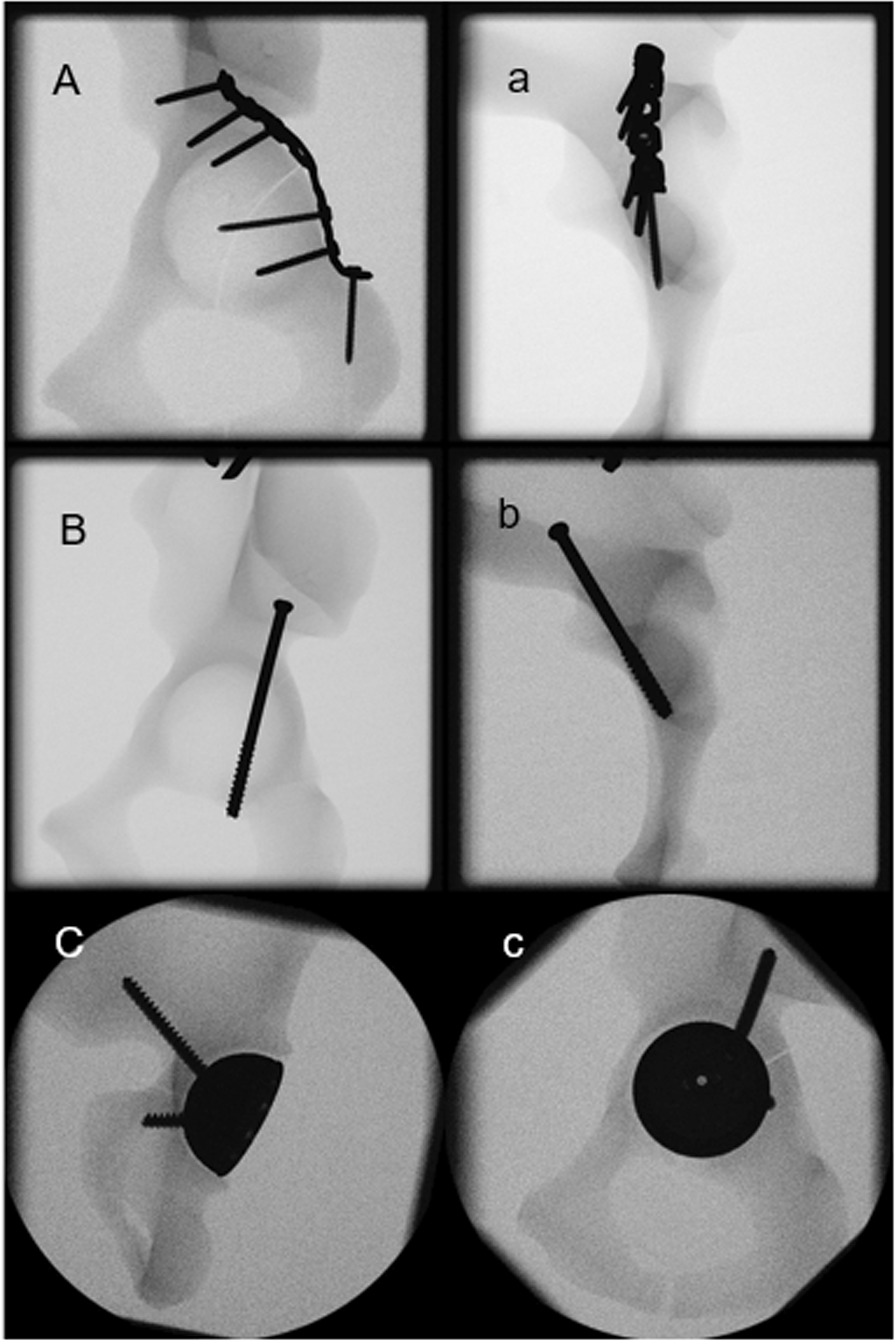
X-rays post instrumentation visualizing exemplified specimens from group PCPF (**A**, **a**), PCSF (**B**, **b**) and PCSC (**C**, **c**)

After instrumentation, anteroposterior and obturator oblique X-rays were performed for documentation and verification of the positioning of the screws in all groups (Fig. 2). Two retro-reflective marker sets were attached to the superior and inferior fragment of each specimen for optical motion tracking.

### Biomechanical testing

**Fig. 3:**
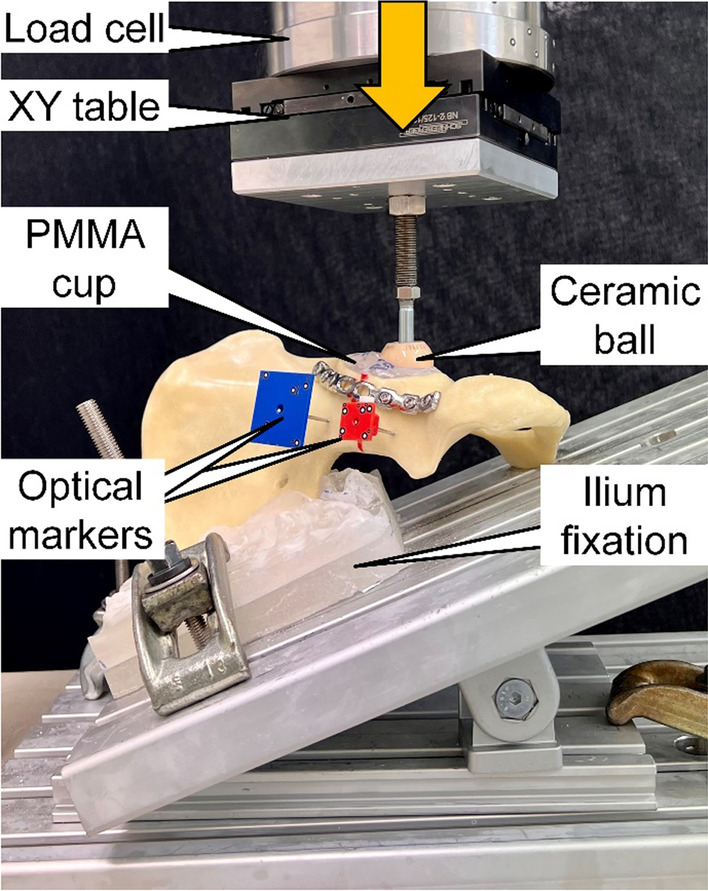
Setup with a specimen mounted for biomechanical testing. Vertical arrow denotes loading direction

Biomechanical testing was performed on a servohydraulic material test system (Mini Bionix II 858; MTS Systems, Eden Prairie, MN, USA) equipped with a 4 kN load cell (HUPPERT 6, HUPPERT GmbH, Herrenberg, Germany). The test setup was adopted from a previous study [18]. Each hemi-pelvis was aligned and tested in an inverted upright standing position. For this purpose, it rested on an aluminum base plate, rigidly secured to the machine base and inclined at 20° in the coronal plane for positioning of both the medial aspect of the symphysis and the sacroiliac joint aspect flush with the base plate according to Morosato et al. [19]. The sacroiliac joint was additionally constrained to the base plate via two molded PMMA blocks, which allowed consistent mounting of all specimens (Fig. 3).

Axial compression along the machine axis was applied to the acetabulum via a ceramic ball of 56 mm diameter. Homogenous load transfer to the specimens was achieved using a molded PMMA hemispherical cavity inserted in the acetabulum. This configuration targeted a simulation of a hip joint reaction force trajectory during walking, as described by Bergmann et al. [20].

The loading protocol commenced with a nondestructive quasi-static ramp from 20 N preload to 200 N at a rate of 18 N/s, followed by progressively increasing cyclic loading in axial compression with a physiological profile of each cycle at a rate of 2 Hz [20]. Keeping the valley load at a constant level of 20 N, the peak load, starting at 200 N, was monotonically increased cycle by cycle at a rate of 0.1 N/cycle until reaching a 10 mm actuator displacement with respect to its position at the beginning of the test. This test stop criterion was considered adequate to provoke catastrophic failure of the specimens [21, 22].

### Data acquisition and analysis

Axial displacement and axial load were continuously acquired throughout testing from the machine transducer and load cell at 200 Hz, respectively. Based on these data, construct stiffness was calculated from the ascending load–displacement curve of the initial quasi-static ramp within the linear loading range between 100 and 160 N.

Furthermore, the coordinates of the optical markers, attached to the specimens, were continuously acquired throughout testing at 20 Hz by means of stereographic optical motion tracking using contactless full-field deformation technology (Aramis SRX, Carl Zeiss GOM Metrology GmbH, Braunschweig, Germany), operating at a resolution of 12 megapixel and maximum acceptance error of 0.004 mm to assess the interfragmentary movements in all six degrees of freedom. Based on the motion tracking data, the following parameters were evaluated: (1) fracture displacement anterior, defined as the translational displacement between the two fragments at the most anterior aspect of the acetabular fracture part; (2) fracture displacement inferior, defined as the translational displacement between the two fragments at the most inferior aspect of the acetabular fracture part; (3) fracture gap opening, defined as the out-of-fracture-plane angular displacement between the two fragments; and (4) fracture gap twisting, defined as the within-fracture-plane angular displacement between the two fragments. The outcome values of these parameters were analyzed after 1000, 2000, 3000, 4000, 5000, and 6000 test cycles under peak loading with respect to the beginning of the cyclic test. The latter number represented the highest rounded number of cycles when none of the specimens had failed so that dropouts could not artifactually affect the results. Reaching 1 mm fracture displacement anterior was arbitrary set as a clinically relevant failure criterion, and the corresponding number of cycles until its fulfillment under peak loading—defined as cycles to failure—was calculated together with the corresponding peak load, defined as failure load.

Statistical analysis was performed with SPSS software package (v.27, IBM SPSS, Armonk, NY, USA). Normality of data distribution was screened and proved with Shapiro–Wilk test. Significant differences among the groups regarding construct stiffness, cycles to failure and failure load were detected with One-Way Analysis of Variance (ANOVA) and Bonferroni post hoc test for multiple comparisons. General Linear Model Repeated Measures test and Bonferroni post hoc test were conducted to identify significant differences among the groups with regard to the parameters of interest evaluated over the time points after 1000, 2000, 3000, 4000, 5000, and 6000 test cycles. Level of significance was set at 0.05 for all statistical tests.

## Results

Construct stiffness (N/mm) was 154.8 ± 68.3 (mean ± standard deviation, SD) for PCPF, 133.3 ± 27.5 for PCSF, and 107.3 ± 41.0 for PCSC, with no significant differences among them, *p* = 0.173.

**Fig. 4:**
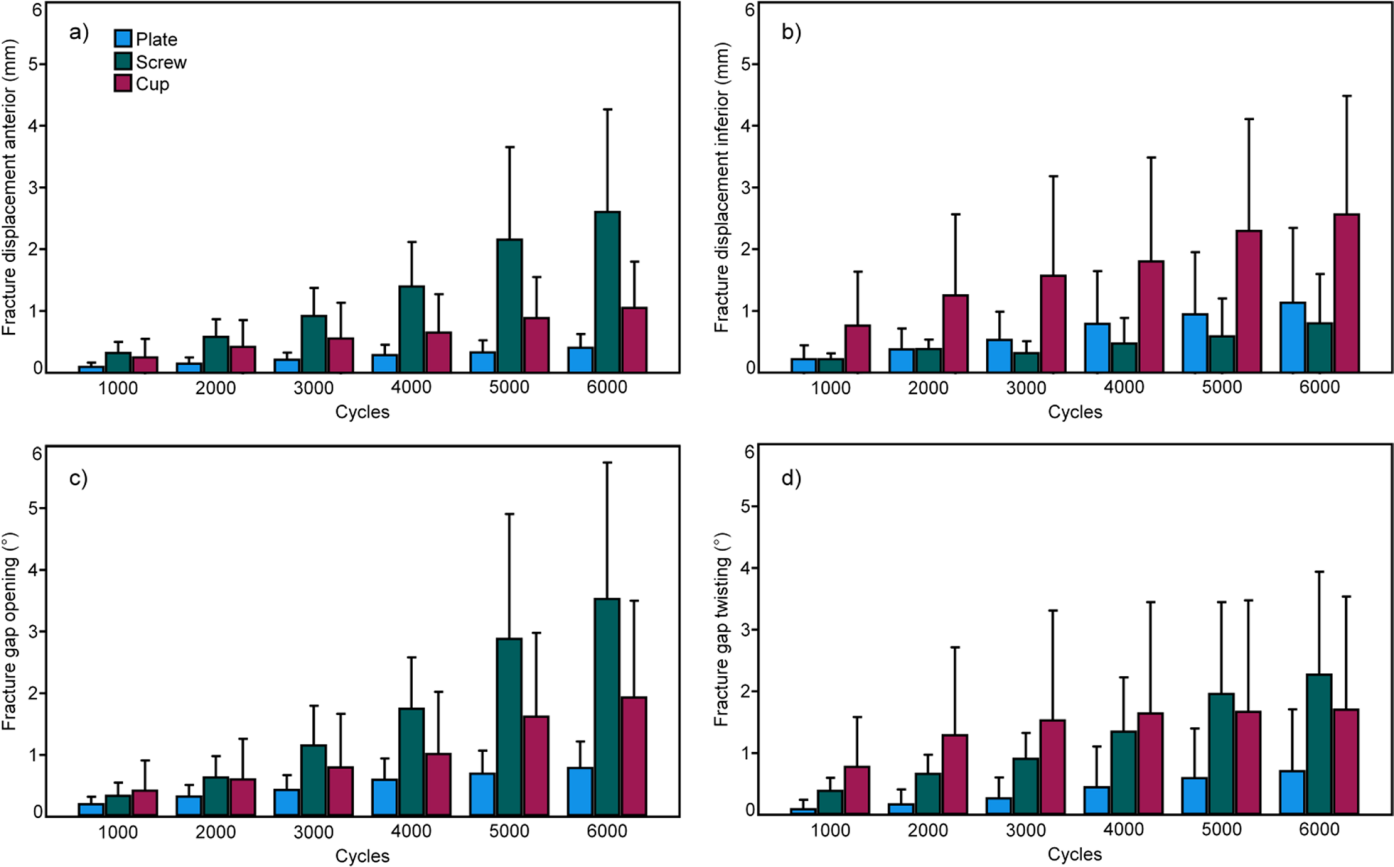
Fracture displacement anterior (**a**), fracture displacement inferior (**b**), fracture gap opening (**c**), and fracture gap twisting (**d**), presented for each separate group PCPF (Plate), PCSF (Screw) and PCSC (Cup) over the five time points after 1000, 2000, 3000, 4000, 5000, and 6000 test cycles in terms of mean and SD

The outcome measures for the parameters of interest evaluated over the five time points after 1000, 2000, 3000, 4000, 5000, and  6000 cycles are summarized in Fig. 4.

Fracture displacement anterior was significantly bigger for PCSF versus both PCPF and PCSC, *p* ≤ 0.049, with no significant differences between the latter two groups, *p* = 0.474. Similarly, fracture gap opening was significantly bigger for PCSF versus PCPF, *p* = 0.016, with no further significant differences between the other pairs of groups, *p* ≥ 0.325. Fracture displacement inferior trended to be higher for PCSC versus PCPF, *p* = 0.054, with no further trends or significant differences between the other pairs of groups, *p* ≥ 0.131. Fracture gap twisting resulted in no significant differences among the groups, *p* = 0.121.

**Fig. 5:**
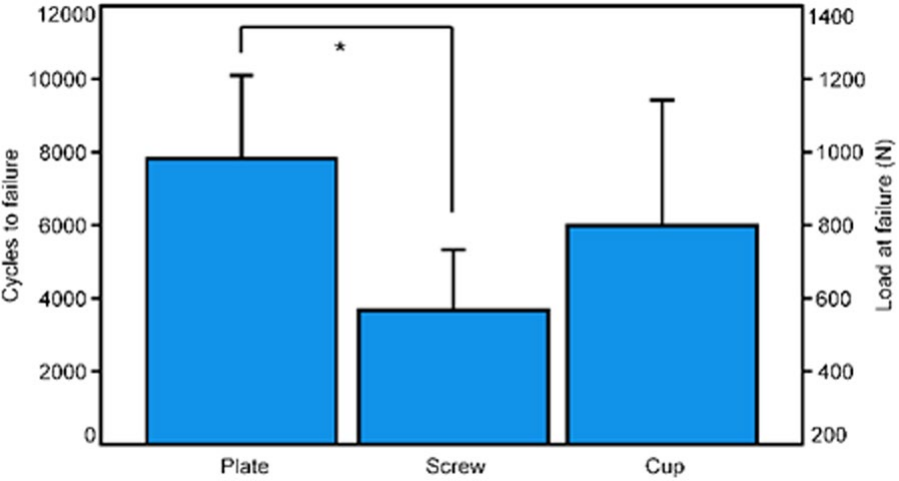
Cycles to failure and corresponding failure load presented for each separate group PCPF (Plate), PCSF (Screw), and PCSC (Cup) in terms of mean and SD. Star indicates significant difference

Cycles to failure and failure load were highest for PCPF (7822 ± 2281 and 982.2 ± 428.1 N), followed by PCSC (5989 ± 3440 and 798.9 ± 544.0 N), and PCSF (3662 ± 1664 and 566.2 ± 366.4 N). Both they were significantly higher for PCPF versus PCSF, *p* = 0.012, with no further significant differences between the other pairs of groups, *p* ≥ 0.253 (Fig. 5). The catastrophic failure modes were characterized by a supraacetabular horizontal fracture in all specimens.

## Discussion

The aim of this biomechanical study was to evaluate primary stability; therefore, a loading scenario simulating walking was considered to evaluate three different methods of posterior column acetabular fracture fixation. With regard to the biomechanical testing results, we identify the following three main important points:

1. Initial construct stiffness was comparable between the three fixation methods.
2. The displacement at the anterior fracture site aspect was significantly bigger following screw fixation versus both plate and cup fixations.
3. Significant differences between the plate and screw fixations were identified in the numbers of cycles to clinically relevant failure and the corresponding failure load.

It has been reported that there is a lack of evidence-based studies regarding the postoperative management of unstable acetabular fractures [23]. Previous biomechanical investigations questioned the justification of restrictive weight bearing [20, 24]. Particularly for geriatric patients, compliance with partial weight bearing can be tremendously more difficult with related negative secondary effects. "The primary aim of operative treatment in elderly individuals is the avoidance of immobilization of the patient" [25]. In these scenarios, cementless acetabular arthroplasty is an acknowledged technique for both primary and revision THA [26–28].

Transacetabular screws are recommended for placement in the dome area of the acetabulum, which provides the best bone stock, while minimizing the risk of iatrogenic damage to surrounding structures [27, 29, 30].

In their groundbreaking work on safe placement of screws in the acetabulum, Wasielewski et al. reported that the posterosuperior and posteroinferior quadrants of the acetabulum incorporate the best bone stock and both regions are relatively safe for transacetabular screw placement [31]. This work represents the foundation of the surgical technique with use of a cementless screwable cup. In the described locations featuring best bone stock, screws for additional support of THA cup can be placed with a good conscience, especially in geriatric patients. With a clockwise rotation of the cup by 45°, the cranial monocortical screw comes to rest in the region of the described safe zone of the superior dome, while the caudal screw can be placed in the posteroinferior quadrant of the acetabulum in a bicortical manner. According to the literature, iatrogenic injuries to blood vessels and nerves from transacetabular screws used in THA are uncommon [32, 33]. Our results demonstrated that the displacement at the inferior fracture aspect trended towards significantly higher values for cup fixation versus plating. There seems to be a weak spot for the inferior screw placement of the cup fixation (in the inferior fracture region), since the fixation of the whole inferior fragment—with fracture line extending into the ischial ramus—seems to be not sufficiently addressed using one screw with a suboptimal orientation with regard to the main fracture plane. Even with regard to initial stiffness, the mean values for plating outperformed those for cup fixation by approximately 50%, which did not reach statistical significance, but may well have a clinical impact. Accordingly, the cup fixation does not seem provide sufficient initial stability as well as stability under dynamic loading, raising the question whether full weight bearing following cup fixation can be recommended, or whether the pressfit principle of cup fixation works at all in case of a fractured acetabulum.

Having a closer look at the principle of screw fixation, in the current study this technique was associated with the highest interfragmentary movements regarding fracture displacement anterior and fracture gap opening, and resulted in earliest fulfillment of the failure criterion, thus qualifying itself as the least stable fixation technique under dynamic loading, despite being associated with an intermediate initial stiffness. This can be interpreted by the characteristic trajectory of screw fixation, which provides a good initial compression when applied perpendicular to a fracture plane, but cannot compensate for rotational and bending forces and moments acting in other directions.

Finally, the plating outperformed both screw and cup techniques regarding all investigated biomechanical parameters, which can be attributed to its multiplanar fixation. Since stiffness reflects the global construct response to loading, it is anticipated that other factors, such as material quality variations, may also have negatively affected the results.

Although the current study presents a biomechanical investigation on artificial bones, the dialogue on the subject of the approach should not be neglected. This is especially true since a minimally invasive treatment of a PCF should be the basic component of future fracture patient care. THA is performed via the anterior minimally invasive surgical (AMIS®) approach as a standard procedure in the hospitals of the authors [34]. Regarding ORIF, posterior acetabular column fractures can be addressed via the posterior Kocher-Langenbeck approach according to the AO surgery guidelines and recommendations [17]. Known disadvantages of this approach are possibly splitting and partial detachments of the gluteus maximus, piriformis and the external rotators. The approach is further known for periarticular heterotrophic ossifications and endangerment of the sciatic nerve [35, 36]. Described disadvantages of the anterior approach in the literature include heterotrophic ossifications and a shallow learning curve besides better clinical outcomes especially in the early postoperative phase [37, 38].

Osteomalacia or manifest of osteoporosis are frequent comorbidities in patients with acetabular fractures, which can lead to early failures of osteosynthesis and therefore to progressing osteoarthritis of the hip joint [39–41]. This seems to be particularly the case regarding minimally invasive alternatives to ORIF of AFs, such as percutaneous screw fixation. Weaver et al. reported that 30% of their patients had a reoperation with THA within two years following ORIF of the acetabulum [41]. Other authors reported 22% to 45% conversion rate following ORIF of acetabulum fractures to THA [7, 42].

The screw fixation in the present work demonstrated significantly higher anterior fracture displacement when compared to both other fixation methods. Additionally, significant differences between plate and screw fixation were detected in the numbers of cycles to clinically relevant failure. On the other hand, screw constructs resulted in the lowest fracture displacement at the inferior fracture site. Consequently, whereas for screw constructs the fracture gap opened more at the anterior site, for cup constructs this gap opening was withershins. In addition, cup fixation was associated with increased movements at the inferior fracture site as well as with regard to fragment twisting. This could be interpreted by the fact that the screw purchase was closer to the anterior fracture site and therefore it was more effective there, or by the orientation of the screws in relation to the main fracture plane. Furthermore, although fracture gap opening was low, the fragments were able to twist to some extent against each other, which contributed to increased fracture displacement inferiorly.

The posterior window for an antegrade screw placement of the posterior column was deliberately chosen, as this approach has, according to the authors' opinion, the best chance to fulfill the requirements for full weight bearing. The screw is hereby inserted perpendicular to the fracture plane which provides reasonable fracture compression and an anatomical reduction of the fracture.

A recent study, following an analysis of 4918 elderly patients with fractures of the hip, concluded that patients with prescribed postoperative weight bearing restrictions have a significantly greater risk of developing more adverse events. Yet, nearly 25% of the orthopaedic trauma surgeons fail to follow this evidence-based guideline [43].

In a recent review article, the optimal therapy using a hip revision cup alone, or implementing THA plus an additional ORIF, or considering a treatment with either a one-step or a two-step approach, could not yet be determined [44]. In addition, the results of the current study could only provide a partial input to the discussion on this topic. Therefore, future studies are needed to generate further knowledge in this field and unambiguously answer the question, possibly taking into consideration new operative techniques too [45].

### Strength and limitations

Main limitation if the current study is the choice of an artificial bone model. Nevertheless, this investigation constitutes an atypical experimental method of full weight bearing following posterior column fracture fixation with little data available in the literature. Accordingly, the authors have decided to perform this study as a first step approach, that justifies ethically the initiation of further cadaveric investigations. It is known that artificial pelvises allow examination in a standardized cost-effective fashion, and that the multitudinous differences in bone quality present in human cadaveric specimens might be overpowered [46–49]. In addition, synthetic bone specimens have been regularly and efficiently used in several biomechanical studies, specifically focusing on the pelvis [48, 50–53]. Moreover, there is a poor availability of cadavers, which can affect the sample size for biomechanical experimentations [54]. Further, the use of artificial bone models minimizes the variability of test results between the specimens [50, 55]. While the chosen sample size was relatively small, it was comparable to equivalent biomechanical studies investigating pelvic fixation techniques [50–53, 56]. Finally, the failure criterion, although arbitrarily chosen, deemed relevant, because in most cases the specimens experienced a sudden drop in stability in close relation to this criterion.

## Conclusion

Standard ORIF of PCF with either plate osteosynthesis or using a screwable cup for THA demonstrated encouraging results for application of a post-surgical treatment concept with a full weight bearing approach. Further biomechanical cadaveric studies with larger sample size should be initiated for a better understanding of AF treatment with full weight bearing and its potential as a concept for PCF fixation.

## Data Availability

The Codes are available upon reasonable request from the corresponding author. The collected data will be stored securely in our institute for 10 years. During this period, they are still available upon request. After 10 years, the data will be deleted; however, all the datasets analyzed or generated during this study will be available from corresponding author upon reasonable request.

## Declarations

### Good clinical practice

All methods were carried out in accordance with relevant guidelines and regulations. This study was performed in line with the principles of the Declaration of Helsinki.
